# Microbial Aetiologic Agents Associated with Pneumonia in Immunocompromised Hosts

**DOI:** 10.4314/ajid.v4i1.55084

**Published:** 2010

**Authors:** Theophilus K C Udeani, Joy Moses, Adanma Uzoechina, Ameh E J Okwori, Chigozie N Okwosa

**Affiliations:** 1 Department of Medical Laboratory Sciences, College of Medicine, University of Nigeria Enugu Campus, Nigeria; 2 Department of Medical Microbiology Federal College of Veterinary & Medical Laboratory Sciences National Veterinary Research Institute, Vom-Jos, Nigeria

**Keywords:** Immunosuppression, Microbial isolates, tuberculosis, HIV, Coinfection

## Abstract

Pulmonary infections are a major cause of morbidity and mortality in the immunosuppressed patients. The aim of this study was to determine the etiologic agents and predisposing factors associated with pneumonia infections in immunocompromised patients. Cross-sectional survey of 100 immunocompromised patients due to HIV and *Mycobacterium tuberculosis* infections were enlisted for the study. The patients completed a structured questionnaire to abstract information on demographic features and risk factors. Sputum samples were collected from the patients with clinical suspicion of having pneumonia and the sputa examined by cultural methods. The tuberculosis patients had the highest number of isolates, 119 (70%) while those with co-infections of HIV/AIDS and tuberculosis had 41(24.1%) and those with only HIV infection were 10 (5.9%). The distribution of isolates were as follows, *Staphylococcus aureus* 63 (37.9%), *Streptococcus pyogenes* 44 (25.9%), *Streptococcus pneumoniae* 27 (15.9%), *Candida albicans* 24 (14.1%), *Klebsiella pneumoniae* 7 (4.1%), *Proteus mirabilis* 4 (2.4%) and *Escherichia coli* 1 (0.5%). Those with previous history of alcoholism and tobacco smoking had relatively high isolates. This study demonstrated that secondary infections are prevalent in the immunocompromised patients due to HIV/AIDS and TB or co-infection with TB/HIV-AIDS. This may lead to drug resistance, DOTS or HAART programme, thereby leading to high mortality and morbidity.

## Introduction

Pulmonary infections are a major cause of morbidity and mortality in immunocompromised patients (Boughman, 1999). Pneumonia is usually triggered when the individuals defense system is weakened as a result of immunoglobulin deficiency (such as multiple myloma), neutrophil defect (neutropenia patient after cancer chemotherapy) or T-cell defect (Transplant or infectious diseases) (Boughman, 1999).

Community-acquired pneumonia affects nearly four million adults each year with *Streptococcus pneumoniae* as the common bacterial agent (Gupta and Sarosi, 2001). Pinner et al. (1996) reported that in USA, between 1979 and 1994, the overall death rates associated with pneumonia and influenza increased by 59%. Annually, 2–3 million cases of community acquired pneumonia results in high rate of hospitalization and more than 45,000 deaths in the USA (CDC, 1997; Marston et al., 1997). The incidence of community-acquired pneumonia requiring hospitalization is estimated to be 258 cases per 100, 000 population and 962 cases 100,000 people's ≥65yr of age (Marston et al., 1997). The mortality of pneumonia has ranged from 2% to 30% among hospitalized patients in a variety of studies; average rate is approximately 14% (Fine et al., 1997). In cases where an individual is immunocompromised, the tendency is that the lower respiratory tract (lungs) will be affected. This will complicate the clinical nature of such individuals. In most cases, these agents (pneumonia) are not routinely sort for in routine laboratories, coupled with poor prognosis in such individuals. It is, therefore, necessary to ascertain the extent of pneumonia causing bacteria in complicating such individuals' health, thereby causing high mortality. In some cases, infections such as tuberculosis which mimics pneumonia are treated as pneumonia cases and vice-versa.

The aim of this study was to determine the bacteria and yeast-like agents associated with pneumonia in immunocompromised hosts and the risks associated with the co-infections.

## Materials and Method

### Study population

The subjects were individuals with immunocompromised health due to the infection with Human Immunodefiency Virus/Acquired Immunodeficiency Syndrome (HIV/AIDS) and *Mycobacterium tuberculosis*. They were patients presenting additional pulmonary infections, either diagnosed by X-ray or by physical examination by the physicians. The study site was a rural hospital, Mangu Cottage Hospital, Jos, Nigeria, dedicated to diagnosis and treatment of this group of patients. Briefly, the patients of all ages were eligible for the survey. After giving their informed consent, the participants were interviewed by using a standard structured questionnaire, in English or in a local language during visits to the hospital. This was to abstract information on demographic characteristics, clinical and behavioral patterns that might expose them to infections.

### Sputum collection and processing

The patients were asked to rinse their mouth with water, and cough-out sputum from the depth of the lungs, for adults; while the parents of the children were instructed to collected the sputa as the child coughs, avoiding the first sputa that was coughed out. The sputum samples were collected in sterile containers, transported to the laboratory for analysis. The quality of the sputum was determined by its consistency and cellular make-up. The sputum samples were inoculated unto brain Heart Infusion Broth (BHI), Tryptose Soya Broth (TSB) and nutrient broth. About 0.05ml of the samples was inoculated directly unto Brain Heart Infusion Agar, blood agar and chocolate agar fortified with 10% sheep red cells respectively; and on Saubrauod dextrose agar. Brain heart infusion agar (without sheep red cells) was used to rule out dimorphic fungi. The samples were inoculated in duplicates (with the exception of SDA). One set was incubated at 37°C, while the second set was incubated at increased 10% CO_2_ tension at 37°C for 24–48 hrs. The inoculums on SDA and BHIA were incubated for up to 7days to check for opportunistic and dimorphic fungi. The suspected Gram-negative pathogens were sub-cultured unto MacConkey agar (MCA), from the broth cultures; and were incubated at 37°C for 18–24 hrs.

### Identification of the isolates

The isolates were identified using conventional bacteriological techniques (Balows and Harsier, 1991) The identification was based on morphological growth characteristics, gram stain, and biochemical testing using OF medium. .Pneumococci identification was based on optochin susceptibility and bile solubility. A definitive diagnosis of the aetiological agent was made when the sputum cultures yielded one or more predominant bacterial strains.

## Results

A total of one hundred immunocompromised patients were recruited for the study. They comprised of 7(7%) HIV/AIDS patients, 17 (17%) TB/ HIV-AIDS patients, 76 (76%) Tuberculosis (TB) patients. The study populations were made up of 42 (42%) males and 58 (58%) females. The age range varied considerably from 3 months to 90 years old, which is a reflection of societal disease distribution.

Out of the hundred sputa samples, 1 (1%) had no isolates, 33(33%) yielded single isolates, 61 (61%) yielded two isolates each while 5(5%) yielded three isolates each. Overall, a total of 170 isolates were identified from 99 (99%) sputum samples. The bacterial isolates were more in tuberculosis patients with 119 (70%) isolates, followed by those co-infected with HIV/AIDS and TB with 41 (24.1%) and the HIV/AIDS patients with 10 (5. 9%) isolates (Table I). *Candida albicans* was found to be the predominant isolates in TB/HIV-AIDS patients, with 10.0% isolates, while in HIV/AIDS and TB patients, the frequency of isolation were 3.0% and 1.2%. *Staphylococcus aureus* had the highest frequency of isolation in TB patients with 28.2% isolates, whereas in HIV and HIV/AIDS-TB, the number of isolates was 7.6% and 1.2% respectively. The isolation of *Streptococcus* species were more in TB patients than in HIV and HIV/TB patients. The frequency of *Streptococcus pyogenes* was as follows; in TB patients 22.9%, HIV/TB 3.0% and no isolates was seen from HIV/AIDS patients. The occurrence of *Streptococcus pneumoniae* in TB patients was 11.2%, 2.9% in HIV/TB patients and 1.8% in HIV patients. The frequency of isolation of *Klebsiella pneumoniae* was more in TB patients with 3.5% and 0.5% in HIV/TB patients and no isolates from HIV patients. *Proteus mirabilis* (2.4%) and *Escherichia coli* (0.5%) were isolated from TB patients only.

**Table 1:** Microbial isolates in relation to demographic features and disease condition

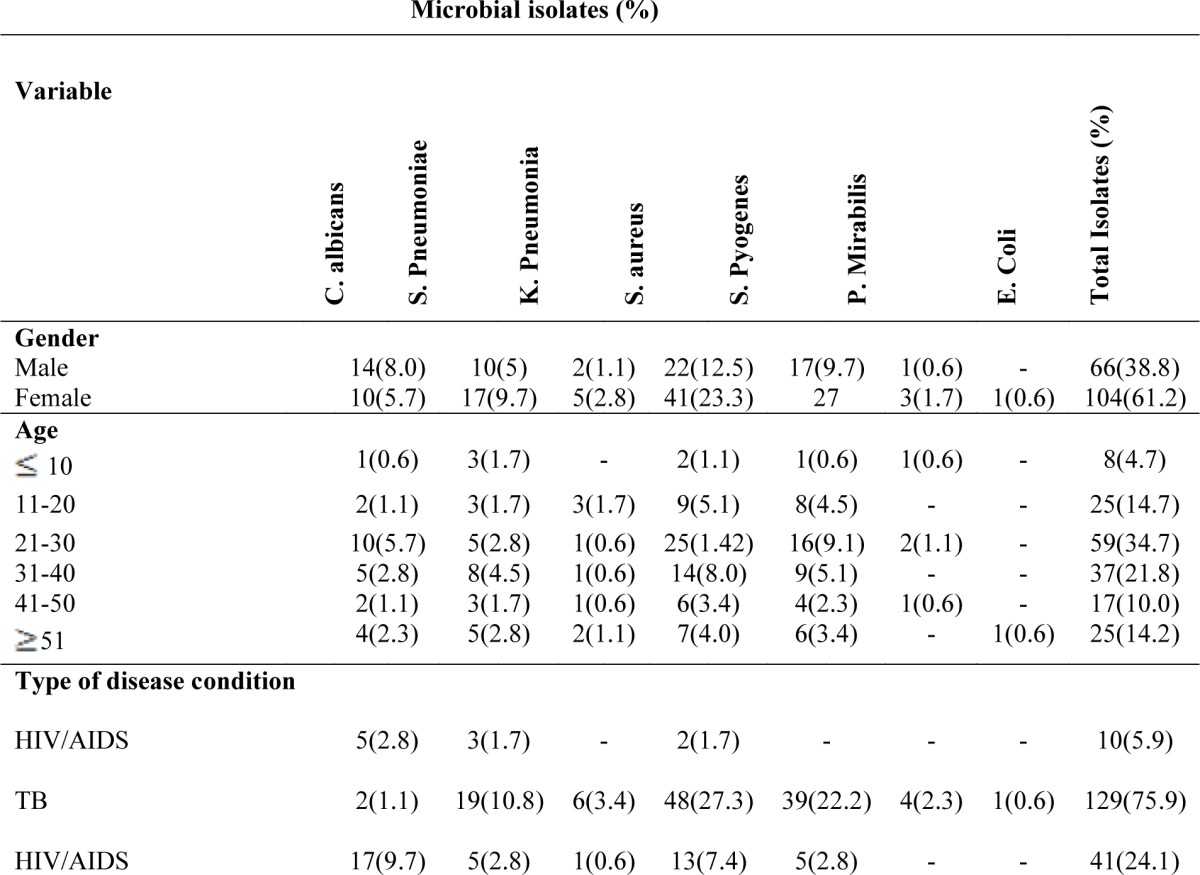

In HIV/AIDS (Table 1), the age group 21–30 had more isolates 10.6% with *Candida albicans* being the highest 4.7% of the isolates followed by age group 31–40 (5.9%) isolates with *Candida albicans* being the highest 3.0% and ≥51 being 4.7% with *Candida albicans* still the highest isolates. In TB patients (Table 1), age group 21–30 had the highest isolates (24.1%) with *Staphylococcus aureus* (11.2%) being the highest of the isolates, followed by age group 31é40 (15.9%) with *Staphylococcus aureus* still the highest isolates (7.1%) and age group 11–20 (11.9%) with *Staphylococcus aureus* being (4.7%) the highest of the isolates. The sex distribution pattern of the isolates (Table 3) showed that in TB patients, females had more isolates (48.8%) with *Staphylococcus aureus* being the highest (18.9%) of the isolates, followed by *Streptococcus pyogenes* (15.3%) and *Streptococcus pneumoniae* (8.8%); whereas in males 22.9% of the isolates were identified, with *Candida albicans* still the highest (4.7%), followed by *Staphylococcus aureus* (3.0%) of the isolates.

Clinical characteristics (Table 2) of the patients showed that those with cough had the highest frequency of isolation (96.5%), while those without cough were 3.5%. Patients with chest pains had frequency of 68.2% whereas those without chest pain had the frequency of 31.8%. Those diagnosed of pneumonia by X-ray had higher frequency of isolates (67.1%) while those not diagnosed by X-ray had a frequency of 32.9%. Patients who were not vaccinated against TB had the highest frequency of isolates (95.3%) while those vaccinated had 4.7% frequency of isolates.

**Table 2:** Clinical and behavioral characteristics of the subjects

Clinical characteristics	N	Frequency of isolates (%)
Cough:		
Yes	97	164 (96.5)
No	3	6 (3.5)
Chest Pain:		
Yes	67	116 (68.2)
No	33	54 (31.8)
Confirmation of pneumonia b y		
X-ray:		
Yes	63	114 (67.1)
No	37	56 (32.9)
Previous alcohol intake:		
Yes	7	10 (5.9)
No	93	160 (94.1)
Previous cigarette smoking habits:		
Yes	5	8 (4.7)
No	95	162 (95.3)

**Duration of infection:**		

< 1 year	81	150 (88.2)
≥ 1 year	19	20 (11.8)

The patients' response to previous history of alcohol consumption was poor as only 7 patients consented to alcohol use. Out of these, 10 (5.9%) bacterial isolates were obtained, while those that had no previous history of alcoholism had 95.3% bacterial isolates. The pattern of previous cigarette smoking was similar to that of alcohol. Out of the 5 respondents who had previous tobacco experience, only 8 (4.7%) bacterial isolates were made, where as those that has not taken tobacco gave bacterial isolates of 162 (95.3%).

## Discussion

The study demonstrated that secondary infections are prevalent in the immunocompromised patients due to tuberculosis (TB) and/or Human Immunodeficiency Virus/Acquired Immune deficiency Syndrome (HIV/AIDS). The main results of the study were that: (1) patients aged 21–30 years old were more likely to have an etiology comprising of typical bacterial pathogens; (2) *Staphylococcus aureus, Streptococcus pyogenes and Streptococcus pneumoniae* were mostly bacterial isolates that complicates *M.tuberculosis* infection, while *C.albicans* was isolated in the majority of HIV-AIDS/TB patients.

The age groups of 21–50 years olds had more bacterial pathogens, with *S.aureus* and *S. pneumoniae* accounted for majority of the isolates. This finding agrees with the report of several other studies (Venkatestan et al., 1990; Riquelime et a.l, 1996). In this study, the patients with co-morbid infection of tuberculosis were associated with *P. mirabilis* (2.4%) and *E. coli* (0.5%). This isuggests that co-morbidity rather than age was the determining condition predisposing to these etiologies Accordingly, these etiologies were reported in two studies of community acquired pneumonia in the elderly, ranging from 0 and 5% (Rosen, 1994; Barrtlet and Mundy, 1995; Lieberman et al., 1996), as infrequent causative agents.

The prevalence rate of 12.9% *Candida albicans* isolated from HIV/AIDS patients conforms to the report that most patients are colonized readily with *Candida albicans* which appears as part of the mouth flora in greater than 80% of HIV positive patients (Riquelime et al., 1996) This may be attributed to lack of mucosal immunity and immune suppressive effects of HIV infection. The low yield of *C. albicans* (1.2%) isolates in TB patients indicates that this yeast is not a defining feature in the progression of TB infection. and may probably be as a result of co-infection of TB with HIV/AIDS.

The encapsulated bacteria, *Streptococcus pneumoniae*, were common etiologic agent in immunocompromised patients. Bartlett and Mundy (1995) identified 5 to 18% *S. pneumoniae* in community-acquired pneumonia as compared to 15.9% in our study. Although, we did not assess the mortality rate, but obviously with the weakened immunity, mortality in these patients will be high. The high frequency *S. pyogenes* isolation (22.9%) from TB patients only, will enhance the progression of lung damage *Klebsiella pneumoniae*, with a low prevalence of 3.5%, can be a co-factor in disease progression of TB infection because it causes chronic lung infections. Thus, TB patients are more likely to have severe disease outcome and high death-rate due to combined effects of *K. pneumoniae* and *M. tuberculosis*. This agrees with the report of Shailaja et al. (2004) that *K. pneumoniae* has devastating effects on the elderly and immunocompromised.

*S. aureus* constitutes 37.1% of the total isolates, being higher in TB patients (28.2%) than HIV/AIDS patients (8.8%). This is higher when compared with the report of Hug and Rossi (2001) with 8.6% isolates of *S. aureus*. This difference can be explained by the fact that Hug and Rossi, (2001) included both Community-acquired pneumonia and immunosuppressed patients where as the co-morbidities in this study, HIV and TB, are known immunosuppressive infectious agents. The frequency of isolation was highest in those harbouring the illness for less than one year of infection. This is probably due to initiation of damage and deterioration of respiratory tract which encourages the influx of bacteria to the airways. The frequency is low in those harbouring illness for one or more than one year probably due to antibiotic chemotherapy which might have flushed out most of the microbes.

Miller and Jones (1964) found that carrier rate of *S. pneumoniae* and *H. influenza* in the nasopharynx was similar for patients with and without chronic sputum production .The present study showed a higher frequency of isolation in patients high cough production than in those without cough. This may be due to high chronicity of the infection and high bacterial load in the lungs due to TB damages, thereby enhancing the multiplication of secondary bacteria infections. Thus all these interactions contribute to the high frequency of cough in these patients. It was also found that the frequency of isolation is highest in patients with chest pain, probably due to damage caused to the chest wall.

The X-ray films of patients with pneumonia show increased fluid and abnormal shadows on the chest wall (Appleyard, 1989; Chong et al., 2006). The patients confirmed of pneumonia despite TB cases by X-ray harbored more isolates which is indicative of pneumonic infiltrates. The analysis of the role of social behavioral patterns of the patients indicates low response to alcohol consumption and cigarette smoking. Despite this, isolates from these patients were relevant to disease outcome. The adverse effects of tobacco smoking on lungs are impaired clearance of bacteria, macrophages inability to kill bacteria (Boughman, 1999). The increased susceptibility to acute and chronic infections among smokers was starkly reflected in cohort studies of mortality from chronic obstructive pulmonary disease, pneumonia and tuberculosis (Doll et al, 1994). The 5.9% of bacterial isolates from those with previous history of alcohol indicates that such patients are at increased risk of pulmonary pneumonia. This is supported by the results of in *vitro* and *in vivo* studies (Anthony et al., 1993) that alcohol abuse favours the development of invasive disease due to decreased activity of alveolar macrophages. One study (Frank et al., 2004) showed a reduction of endotoxin-neutralizing capacity and reduced titers of antilipopolysaccharide antibodies in alcoholic patients thereby rendering alcoholic persons more prone to infections.

In immunocompromised hosts, there is a very high rate of morbidity and mortality due to infectious diseases. The control of these secondary infections will ultimately prolong the life of those infected with either HIV or TB. It is thereby suggested that the mechanism by which an organism colonizes a particular patient more than the other requires further investigations. For instance, in this study; *C. albicans* was found to colonize only HIV patients while *K. pneumoniae* colonized only TB patients, therefore. such disease specific co-infections should be investigated further, together with patterns of drug action.
